# The antagonistic *Metschnikowia andauensis* produces extracellular enzymes and pulcherrimin, whose production can be promoted by the culture factors

**DOI:** 10.1038/s41598-021-89982-y

**Published:** 2021-05-19

**Authors:** Enikő Horváth, Lívia Dályai, Erna Szabó, Teréz Barna, László Kalmár, József Posta, Matthias Sipiczki, Hajnalka Csoma, Ida Miklós

**Affiliations:** 1 Department of Genetics and Applied Microbiology, Faculty of Natural Science and Technology, University of Debrecen, Egyetem tér 1, 4032 Debrecen, Hungary; 2 Department of Ecology, Faculty of Natural Science and Technology, University of Debrecen, Egyetem tér 1, 4032 Debrecen, Hungary; 3 Department of Landscape Protection and Environmental Geography, Faculty of Natural Science and Technology, University of Debrecen, Egyetem tér 1, 4032 Debrecen, Hungary

**Keywords:** Microbiology, Physiology

## Abstract

Biological control against microbial infections has a great potential as an alternative approach instead of fungicidal chemicals, which can cause environmental pollution. The pigment producer *Metschnikowia andauensis* belongs to the antagonistic yeasts, but details of the mechanism by which it inhibits growth of other microbes are less known. Our results confirmed its antagonistic capacity on other yeast species isolated from fruits or flowers and demonstrated that the antagonistic capacity was well correlated with the size of the red pigmented zone. We have isolated and characterized its red pigment, which proved to be the iron chelating pulcherrimin. Its production was possible even in the presence of 0.05 mg/ml copper sulphate, which is widely used in organic vineyards because of its antimicrobial properties. Production and localisation of the pulcherrimin strongly depended on composition of the media and other culture factors. Glucose, galactose, disaccharides and the presence of pectin or certain amino acids clearly promoted pigment production. Higher temperatures and iron concentration decreased the diameter of red pigmented zones. The effect of pH on pigment production varied depending of whether it was tested in liquid or solid media. In addition, our results suggest that other mechanisms besides the iron depletion of the culture media may contribute to the antagonistic capacity of *M. andauensis*.

## Introduction

Species capable of biological control have received particular attention, because they are able to inhibit the growth of other microbes and thereby are able to protect fruits or vegetables against decay^1–3^. Different mechanisms, such as secretion of cell wall-degrading enzymes or other antifungal compounds have been proposed as being responsible for their antagonistic activity^1,3–5^. A further possible method of antagonism is the production of ferric ion-specific chelating agents, which results in iron depletion in the environment and causes growth inhibition of microbes that require iron for their cellular processes^6,7^.

Several *Metschnikowia* species, including the well-known *M. pulcherrima*, are able to produce an iron binding red pigment^8–11^, which is probably closely linked to its antagonistic capacity, given that pigmentless *M. pulcherrima* mutants lack the antifungal activity^11,12^. The red pigment is called pulcherrimin, which is a water-insoluble complex^13^. The complex is formed from pulcherriminic acid and ferric ions, after conversion of cyclo-leucyl-leucyl to pulcherriminic acid^13–15^. Although pulcherrimin has long been known and some genes involved in its synthesis have also been revealed, the details and conditions of pigment synthesis, and the role of pigment production are not entirely clear^12,16^. The pigment might act as a siderophore, although some of its features seem to differ from those of typical siderophores^11,16,17^. Furthermore, pigment production can depend on culture conditions^8^ and this can strongly influence the antagonistic ability of a strain. Besides, it cannot be ruled out either that other processes may also be implicated in the antimicrobial capacity of the *Metschnikowia* species. Specifically, certain *Metschnikowia* species can also produce extracellular enzymes or volatile componds etc.^17^.

A promising *Metschnikowia* species is the less-known *Metschnikowia andauensis,* which produces pulcherrimin^18–21^. This species also has a great potential for biocontrol. Strains of *M. andauensis* have been evaluated for their biological control activity against the most important postharvest pathogens of fruits, like molds *Penicillium expansum*, *P. digitatum*, *P. italicum*, *B. cinerea* and *Rhizopus stolonifer*, *R. oryzae*, *Alternaria alternata* and *Verticillium cinnabarinum* or against fungi involved in crop and/or food spoilage (*Saccharomyces cerevisiae*, *Wickerhamomyces anomalus* and *Dekkera bruxellensis*) and *M. andauensis* was shown to inhibit growth of these species^20–22^.

In order to expand our knowledge of the antagonistic capacity of *Metschnikowia* species, we investigated the biocontrol capacity and pigment production of *M. andauensis.* We hypothesized that these features are similar to the *M. pulcherrima’*s properties. Based on this, we wanted to determine the most important environmental factors which can contribute to the stronger pigment production of *M. andauensis*. Besides, we studied the growth inhibitory effect of the precursor molecule of the pigment and the extracellular enzyme production capacity of *M. andauensis*.

Our results confirmed the biocontrol capacity of *M. andauensis* and revealed that other yeasts living on fruits or flowers can be inhibited by this species. We demonstrated that the red pigment produced by *M. andauensis* is indeed the iron-containing pulcherrimin and explored culture factors that can influence the production and the localisation of the pigment. Our data also indicate that mechanisms other than iron depletion of the culture media can also contribute to the antagonistic capacity of *M. andauensis*.

## Results

### *M. andauensis* is able to inhibit growth of different yeast species

To expand the list of the species which can be inhibited by *M. andauensis* cells*,* wild yeasts isolated from fruits and flowers were investigated. Their taxonomic positions were determined by PCR and sequencing methods, as detailed in Table 1.

**Table 1:** Strains used in this study.

Collection number	Species	Origin	Isolation source
11-1120	*Metschnikowia andauensis*	HA 1657^T^, CBS 10809^T^, 1	
11-578	*Metschnikowia pulcherrima*	CBS5833^T^, ATCC 18406T	
11-11	*Metschnikowia pulcherrima*	CBS 610^NT^, ATCC 22032^NT^	
11-1158	*Metschnikowia crysoperlae*	CBS9803^T^	
11-482	*Metschnikowia koreensis*	Borneo, Brunei	Flower
11-524	*Metschnikowia laotica*	Laos, Luang Prabang, 2	Fallen fruit
11-1062	*Metschnikowia pulcherrima*	Georgia, Tbilisi	Fruit
10-642	*Saccharomyces cereviaise*	S288c, ATCC 204508	
11-481	*Saccharomyces cerevisiae*	Philippines, Manila	Fallen fruit
11-504	*Candida intermedia*	Laos, Luang Prabang	Flower
2-1366	*Candida magnifica*	Syria, Palmyra	Flower
11-493	*Candida orthopsilosis*	Philippines, Manila	Fallen fruit
11-509	*Candida suratensis*	Laos, Luang Prabang	Fruit
11-510	*Candida suratensis*	Laos, Luang Prabang	Fruit
11-465	*Candida stigmatis*	India, Hyderabad, CBS 12699T,3	Flower
11-1055	*Candida verbasci*	Georgia, Tbilisi, CBS 12699T,4	Verbascum flower
11-1193	*Candida insectorum*	Palau, Ngerekebesang	Flower
11-513	*Candida butyri*	Laos, Luang Prabang	Flower
11-484	*Candida boidinii*	Borneo, Brunei	Flower
11-475	*Issatchenkia terricola*	Borneo, Brunei	Lemon
11-486	*Pichia dorogensis*	Borneo, Brunei	Fallen fruit
11-1135	*Pichia kluyveri*	Guatemala, Tikal	Fallen fruit
11-1071	*Starmerella caucasica*	Azerbaijan, Baku, CBS12650T,5	Flower
11-1127	*Trichosporon asahii*	Guatemala, Tikal	Fallen fruit
1	*Botrytis cinerea*	Hungary, Tarcal	

*CBS* CBS-KNAW culture collection, *ATCC* American Type Culture Collection.

1. Molnar and Prillinger 2005.

3. Sipiczki 2010.

4. Sipiczki 2013.

2. Sipiczki 2014.

5. Sipiczki 2013a.

**Figure 1:**
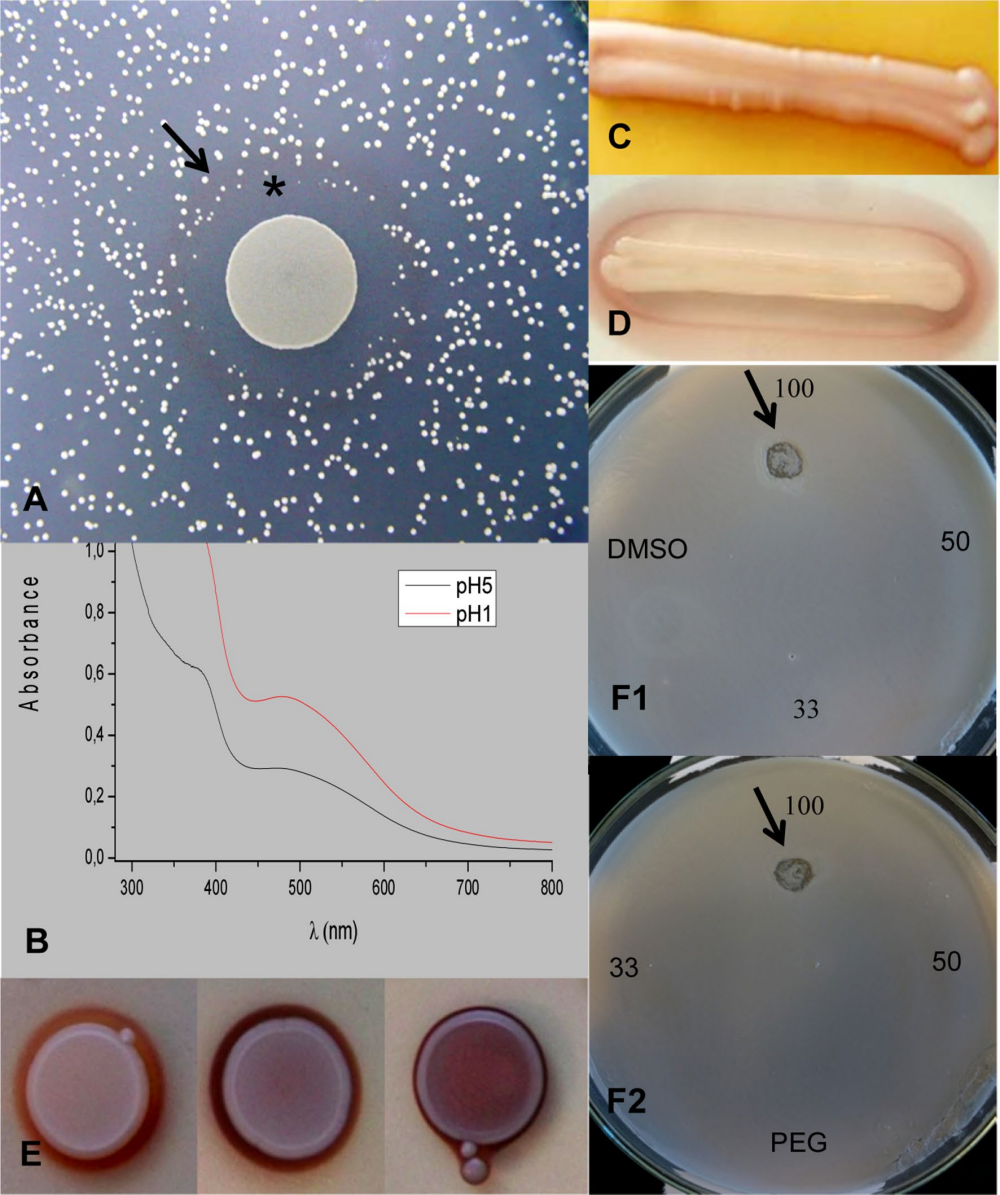
*M. andauensis* is able to inhibit growth of yeasts and produces pulcherrimin. The inhibitory zone of *M. andauensis* on a *Candida magnifica* (11–1366) lawn—indicated with black *—correlated with the red ring formed around the *M. andauensis* cells—indicated with black arrow (**A**). Similar results were obtained on lawns prepared from several other species). The UV–Vis spectrum of the red pigment revealed absorption maxima at 380 and 480 (**B**). The localisation and colour intensity of the red pigment can depend on the composition (**C**, **D**) and iron content of the medium (**E**). (**C**) YPA, (**D**) PDA + 0.001 mg/ml FeCl_3_, (**E**) EMMA, EMMA supplemented with 0.05 and 1 mg/ml FeCl_3_ (from left to right). Growth inhibition of *Starmerella caucasica* (11–1071) by cyclo-leucyl-leucyl (**F**). The cyclo-leucyl-leucyl solution was diluted with DMSO (**F1**) or PEG 4000 (**F2**). Black arrows show the inhibited zone, where the cells were not able to grow compared to the other regions of the agar plates (similar results were obtained in the case of *Candida stigmatis* (11–465) and *Saccharomyces cerevisiae* lawn (10–642). Concentrations of the cyclo-leucyl-leucyl were 100, 50 or 33 mg/ml. PEG and DMSO indicate that PEG 4000 and DMSO were dropped without cyclo-leucyl-leucyl.

Our data confirmed the antagonistic capacity of *M. andauensis,* as it was able to inhibit the growth of several *Candida* species, including *Candida butyri* and *Candida orthopsilosis,* as well as *Starmerella caucasica, Pichia dorogensis, P. kluyveri, Issatchenkia terricola* and *Metschnikowia laotica* (Table 2, Fig. 1A). During these experiments we also noticed that a red pigmented ring often appeared around *M. andauensis* cells and that this red ring correlated with the border of the inhibitory zone (Fig. 1A).

**Table 2:** *Metschnikowia andauensis* can inhibit growth of wild yeast.

Strains used as lawn		Test-strain *Metschnikowia andauensis* (11-1120)			
Collection number	Species	SMA medium			
		pH5	pH7	pH5	pH7
		25 °C		37 °C	
11-504	*Candida intermedia*	−	−	++	++
2-1366	*Candida magnifica*	−	−	+++	+++*
11-493	*Candida orthopsilosis*	++	++	−	−
11-509	*Candida suratensis*	++	++	++	++
11-510	*Candida suratensis*	++	++	++	++
11-1055	*Candida verbasci*	++	++	nd	nd
11-1193	*Candida insectorum*	+	+++	−	+
11-513	*Candida butyri*	++	++	wg#	wg#
11-484	*Candida boidinii*	++	++	nd	nd
11-465	*Candida stigmatis*	++	++	++	++
11-475	*Issatchenkia terricola*	+	+++	−	+++
11-482	*Metschnikowia koreensis*	−	−	nd	nd
11-524	*Metschnikowia laotica*	++	++	nd	nd
11-1062	*Metschnikowia pulcherrima*	−	−	−	−
11-11	*Metschnikowia pulcherrima*	−	−	−	−
11-486	*Pichia dorogensis*	nd	+++	nd	+++
11-1135	*Pichia kluyveri*	−	++	++	++
11-1071	*Starmerella caucasica*	++	+++	+++	+++
11-481	*Saccharomyces cerevisiae*	−	−	−	−
11-1127	*Trichosporon asahii*	−	−	−	−

+ : presence of the inhibitory zone (the number of + indicates the degree of inhibition).

**−** : absence of the inhibitory zone.

nd: inhibition was not determined because of the weak growth of the lawn.

wg#: weak growth of *M. andauensis* on the given lawn.

*See Fig. 1A.

### The *M. andauensis* pigment contains iron and shows the same characteristics as the pulcherrimin

Since *M. pulcherrrima* is able to produce a red colour iron chelated pigment, called pulcherrimin*,* and this was also assumed for *M. andauensis*^18,19^, we decided to examin its pigment. Therefore, the red pigment was isolated from the cell-free supernatant of the *M. andauensis’*s culture medium and was characterized by its UV–Vis spectroscopy. Its iron content was investigated by Flame Atomic Absorption Spectroscopy (FAAS). The UV–Vis spectrum of the *M. andauensis* pigment showed two characteristic absorption bands with absorption maxima at 380 and 480 nm, similar to the spectrum of pulcherrimin produced by *Candida pulcherrima*^13^(Fig. 1B). FAAS analysis showed that the isolated pigment was an iron containing one with 2.19 mg/g iron content.

### Effect of carbon source on pulcherrimin production

Both the localisation and colour intensity of the red pigment varied with different media. To reveal the factors which influence the pigment formation, the *M. andauensis* cells were cultured on media prepared with different components and supplements. The red pigment was produced even in the absence of other microbes and its colour intensity and localisation were strongly influenced by the composition (Fig. 1C,D) and iron content of the media (Fig. 1E). On PDA media supplemented with iron, the red pigment accumulated around the growth, while the yeast cells remained white (Fig. 1D) (no red ring was visible on PDA without iron supplementation). On YDP agar, the cells were pinkish (compared to the colour of the streak grown on PDA) and a pigmented zone around the cells was not observed (Fig. 1C). Furthermore, the localisation and the intensity of the pigmented zone strongly depended on the concentration of Fe^3+^ cations. The increasing concentration of iron resulted in a darker and narrower ring (Fig. 1E—the iron concentration increased from left to right).

**Figure 2:**
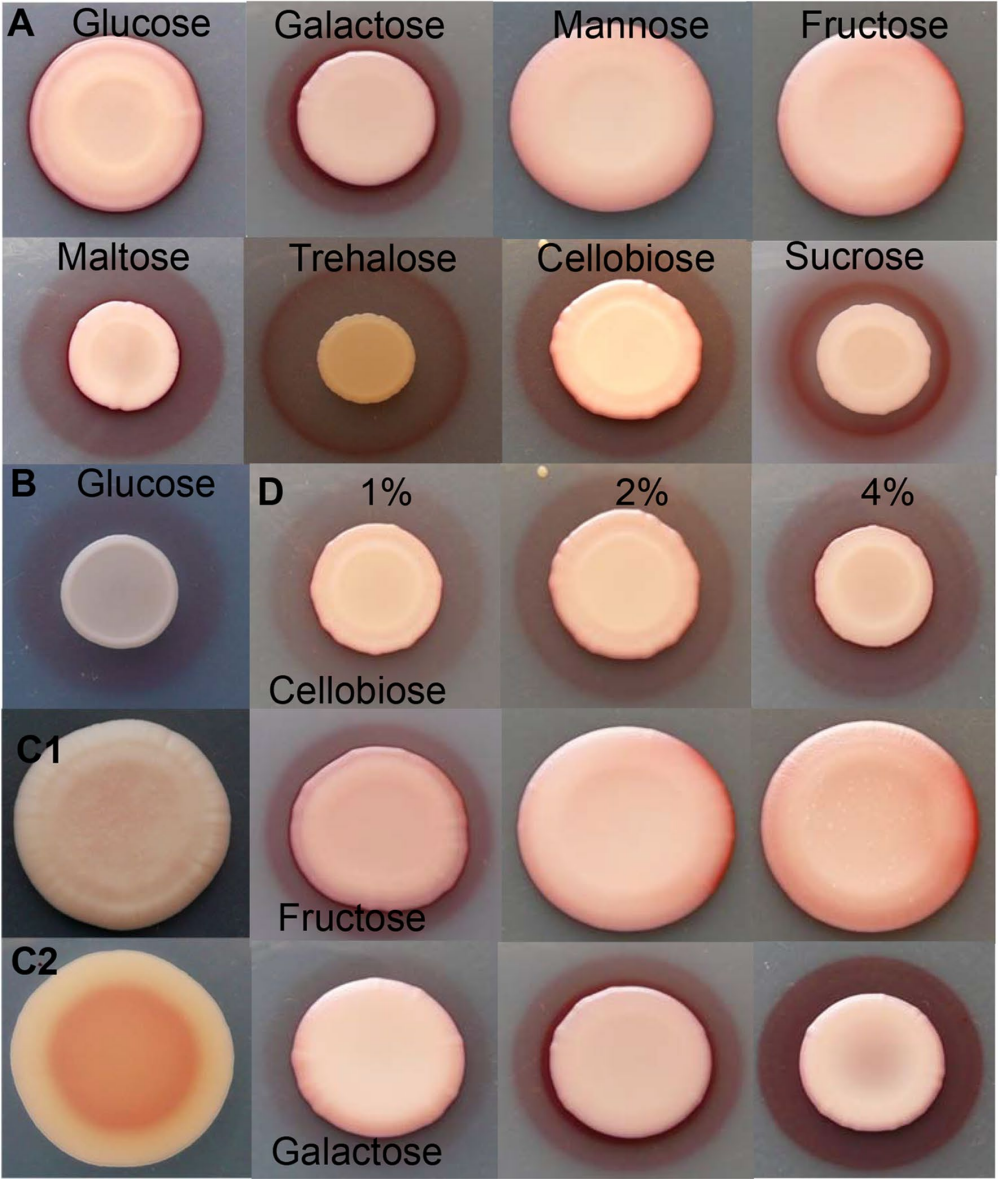
Pigment production can depend on the type and concentration of sugar. SMA contained 2% sugar and 0.001 mg/ml FeCl_3_ and was photographed after 13 days (**A**) and 3 days (**B**). *M. chrysoperlae* produced a small amount of red pigment on 2% maltose containing SMA (**C1,2**). Top side (**C1**) and bottom side (**C2**) of the colony (almost similar results were found on 2 and 4% glucose, sorbose, fructose-containing SMA, 4% sucrose, galactose containing SMA). Pigment production on 1, 2, 4% sugar-containing media (photographed after 13 days) (**D**). (Glucose, maltose, trehalose-containing media were similar to the cellobiose containing media). Mannose-containing medium was similar to the fructose containing media, while sucrose containing medium was similar to the galactose containing medium). Agar plates were incubated at 25 °C.

To clarify the correlation between the compostion of the media, culture conditions and pigment zone formation, *M. andauensis* cells were grown in media prepared from different carbon sources. Specifically, the mono- and disaccharides used as carbon sources in the media highly influenced the size of the pigmented zone (Fig. 2A). Glucose, galactose and the disaccharides maltose, trehalose, cellobiose and sucrose clearly promoted the appearance of large reddish pigmented zones (Fig. 2A,B). However, in the case of glucose, the red pigmented zone, which had appeared after 3 days, was covered by the cells after a longer incubation time (13 days) (Fig. 2A,B). In addition, mannose, fructose, or sorbose did not favour the development of a wide reddish zone around the cells.

The sugar concentration in the media also affected pigment formation by *M. andauensis*, as can be seen on Fig. 2D. Higher concentrations of cellobiose and galactose resulted in larger or darker pigmented halos (glucose, maltose, trehalose and sucrose gave similar results). In contrast, fructose promoted prolonged growth of *M. andauensis* instead of pigment production, as evidenced by the 13-days-old culture covering the reddish halo that had appeared after 3 days (Fig. 2D).

**Figure 3:**
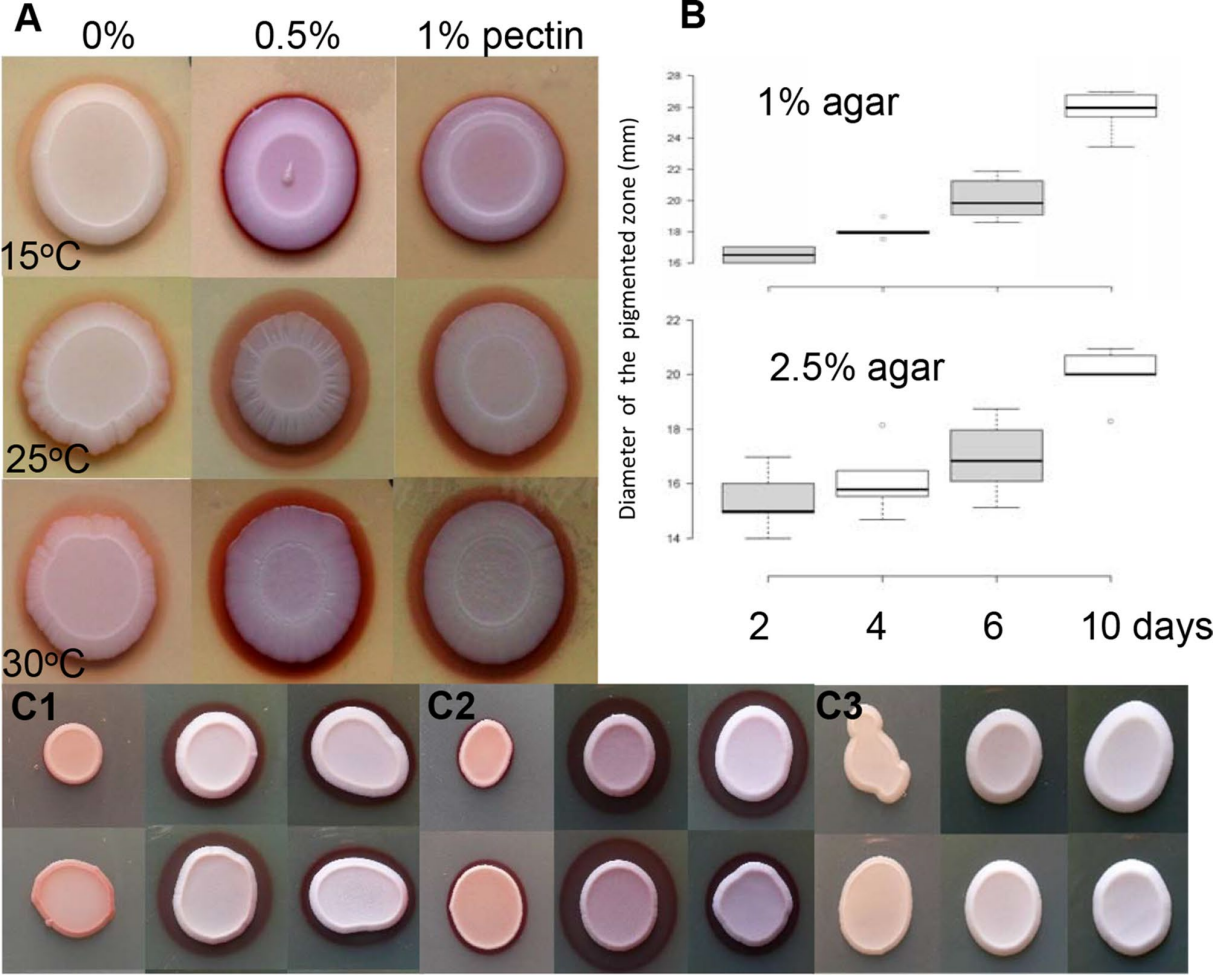
Effect of polysaccharides on pigment production. The presence of the pectin favoured production of a dark red pigmented zone (**A**), while agar influenced rather localisation of the pigment (**B**) and not the pigment intensity (**C**). Size of the pigmented zones on YPA containing 1 and 2.5% agar (6-6 Petri dishes were measured after 2, 4, 6, 10 days. Statistical analysis was created by BoxPlotR program (**B**). SMA, YEA YPA media (from right to left) were incubated at 25 °C and photographed after 5 days (**C1,2,3**). Upper line contained 1.5% agar, bottom line contained 2.5% agar. (**C1**) *M. andauensis* (11–1120), (**C2**) *M. pulcherrima* (11–578), (*M. pulcherrima* (11–11) gave similar results), (**C3**) *M. crysoperlae* (11–1158)*.*

The effects of some polysaccharides on pigment production were also investigated. First, pectin was chosen, as it is an important cell wall component of fruit, and compared with two concentrations of the agar used for the solidification of the media. As Fig. 3A shows, the presence of pectin favoured the production of a darker red pigmented zone at all temperatures, compared to the control (0% pectin). In contrast, the higher agar concentration (2.5%) reduced the size of the halo (Fig. 3B), but did not influence the colour intensity of the pigment (Fig. 3C1). Similar results were obtained in the case of the *M. pulcherrima* strains used as a control (Fig. 3C2).

*M. chrysoperlae,* which was regarded as a non-pigment producing species^32^ and was used as a negative control in these experiments (Fig. 3C3), was also able to produce a small amount of pigment, especially on medium containing 2 or 4% maltose (Fig. 2C1). However, pink cells could be found mainly on the bottom side of the colony and only after 13 days (Fig. 2C2). Almost similar results were found on 4% glucose, sorbose, fructose, sucrose, galactose containing minimal medium (SMA).

### The size of the pigmented halo depends on pH, temperature, the presence of certain amino acids and the cell density

**Figure 4:**
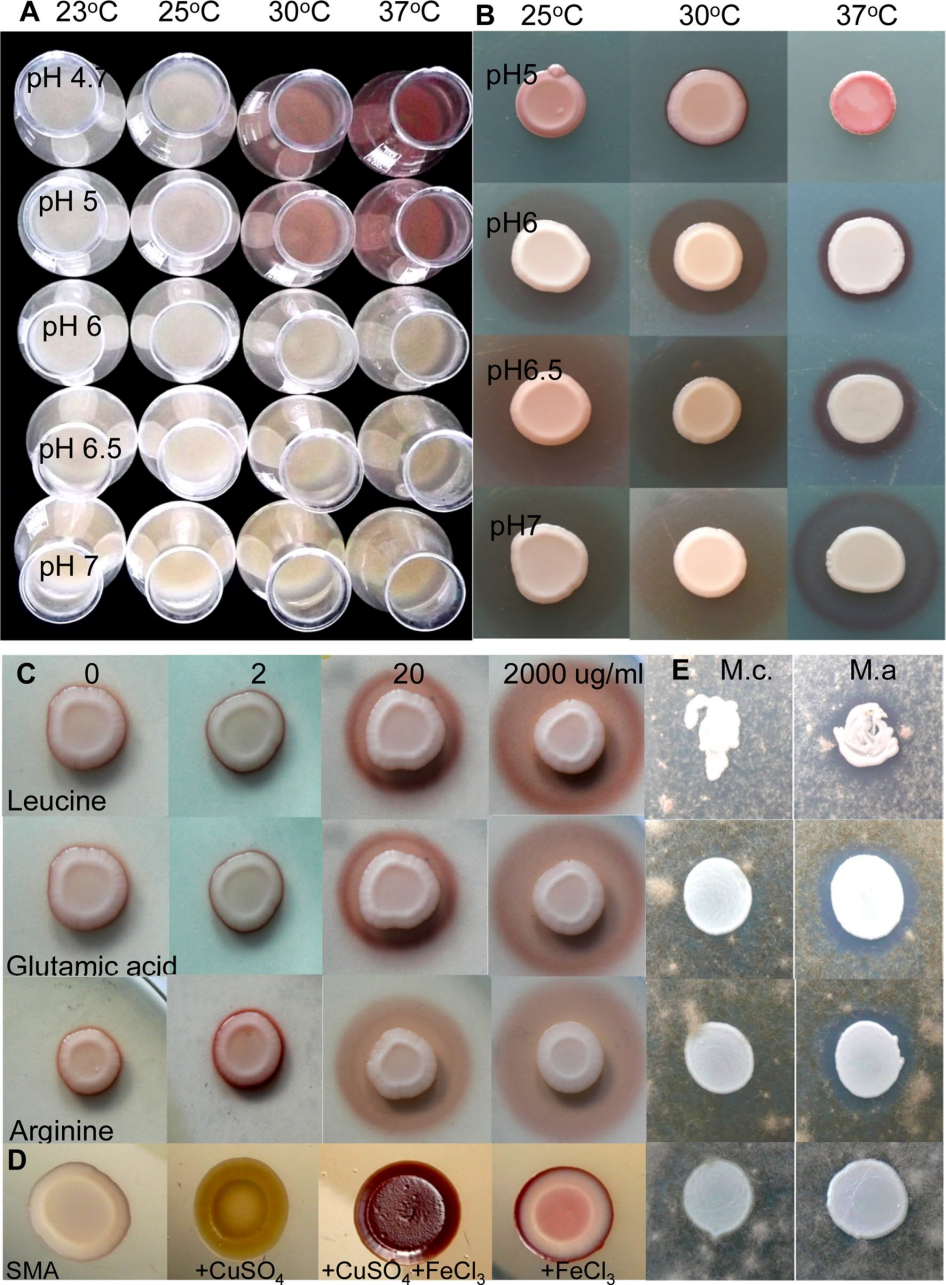
Colour intensity and localisation of the red pigment produced by *M. andauensis* were influenced by culture factors. Lower pH and higher temperature resulted in darker red pigment in liquid medium (**A**) (PDB liquid medium + 0.01 mg/ml FeCl_3_, which were supplemented with 250 µl FeCl_3_ (from 10 mg/ml stock solution) on the 2nd and 3rd days). The cultures were incubated on a shaker for 3 days. The size of the pigmented area was larger at higher pH on PDA (PDB solidified with 1.5% agar and supplemented with 0.01 mg/ml FeCl_3_) (**B**). The presence of amino acids increased pigment production (**C**). (PDA + 0.005 mg/ml FeCl_3_, pH7 after 6 days, incubated at 25 °C. (SMA medium gave a similar result). (**D**) The addition of copper sulphate allowed budding and pigment production to take place. (SMA was supplemented with FeCl_3,_ or Cu SO_4_ or FeCl_3_ plus CuSO_4_). (**E**) Growth of *B. cinerea* in the presence of *M. andauensis* on SMA and SMA supplemented with different amount of CuSO_4_ (from top to bottom: SMA, SMA + 0.0005, 0.005, and 0.05 mg/ml CuSO_4_). The plates were incubated for 5 days at 25 °C. Mc*.*: *Metschnikowia crysoperlea*, Ma: *Metschnikowia andauensis. B. cinerea* was used as lawn.

The effect of further parameters on pigment production, such as incubation temperature, pH of the medium, presence of amino acids and cell density were also tested. Experiments carried out with liquid media revealed that a lower pH and a higher temperature favoured accumulation of the red pigment (Fig. 4A), in contrast to the experiments on solid media, which showed that the size of the pigmented area was larger at a higher pH (Fig. 4B).

The media set to pH 7.0 allowed us to test the relationship between cell density and size of the pigmented halo. A larger pigmented zone was achieved when the initial concentration of the cell suspension was higher. A 15 μl inoculum containing 1 × 10^3^ cells/ml produced a 1.2 mm zone on SMA and a 1.6 mm zone on PDA, whereas a 15 μl inoculum with a density of 1 × 10^7^ cells/ml gave rise to a 2.8 mm zone on both SMA and PDA media (the agar plates were incubated for 5 days at 25 °C).

Figure 4C demonstrates that the presence of leucine, glutamic acid and arginine in the media could strongly increase the size of the pigmented zones compared to media prepared without amino acid supplementation (similar results were obtained with lysine, serine, threonine, alanine).

### Synthetic cyclo-leucyl-leucyl, which is a precursor of pulcherrimin formation, showed growth inhibition on yeasts

The question arose whether growth inhibition can be caused by depletion of iron from the environment alone or also by the presence of cyclo-leucyl-leucyl, the precursor of pulcherrimin formation. To answer this question, cyclo-leucyl-leucyl was synthesized from leucyl dipeptide and its antifungal capacity was investigated using spot assay tests on lawns of *Starmerella caucasica* (11-1071), *Candida stigmatis* (11-465) or *Saccharomyces cerevisiae* cells (11-481, 11-642) (Table 2). Cyclo-leucyl-leucyl caused growth inhibition at the highest (100 mg/ml) of three concentrations (Fig. 1F1,F2).

### The presence of copper ions allows budding and pigment production in *M. andauensis*

Copper sulphate is extensively employed in agriculture because of its fungicidal and bactericidal properties. Furthermore, application of Cu-based chemical treatments is allowed even in organic wineyards. Thus, we wanted to explore the effect of the copper ion on budding and pigment production of *M. andauensis*. Experiments carried out on Cu-containing media showed that the presence of 0.05 mg/ml copper sulphate in the media allowed both pigment production and budding in *M. andauensis* (Fig. 4D), but decreased the size of the inhibitory zone on *Botrytis cinerea* lawn (Fig. 4E).

### *M. andauensis* cells produces various extracellular enzymes

**Figure 5:**
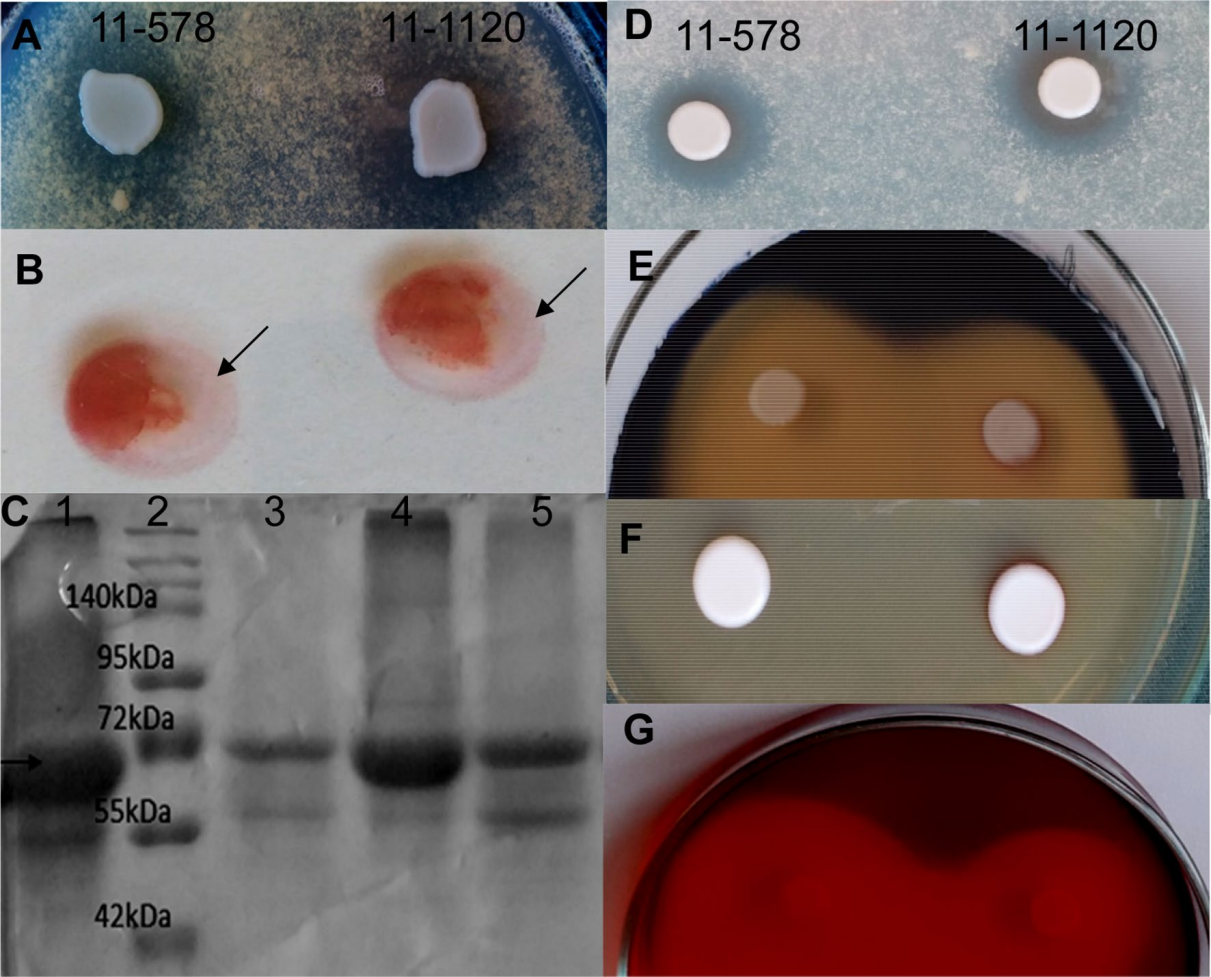
Protease and extracellular enzyme activity of the *M. andauensis* (11–1120) and *M. pulcherrima* (11–578) strains. A, B, C show protease activity: (**A**) clear zones were formed around the *M. andauensis* cells on the casein-containing medium, (**B**) *M. andauensis* cells were able to melt the SMA medium solidified with gelatin. Black arrows show the melted parts of the medium around the *M. andauensis* colonies. (**C**) 12% SDS-PAGE analysis indicated the extracellular protease activity of the *M. andayensis* on BSA. Weaker BSA band was found in the sample 3, compared to the sample 1, which indicates significant protease activity. Sample 1: BSA standard; Sample 2: Protein marker; Sample 3, 4 and 5: Sephadex G-25 chromatographic fractions of the medium. (Sample 3: fraction with 2.5 µml elution volume, Sample 4: fraction with 3.5 ml elution volume; Sample 5: fraction with 5.5 ml elution volume). Black arrow shows the BSA band. D-G prove extracellular activities of the cells. (**D**) Acid- (**E**) amylase- (**F**) β-glucosidase production. (**G**) The lighter red zone around the cells suggests partial degradation of carboxymethyl-cellulose (the plates were incubated at 25 °C).

Given the ability of *M andauensis* to inhibit different yeast species (Table 2), we wondered if other processes besides pulcherrimin production and iron depletion might also contribute to the antagonistic activity of this species. To this end, the extracellular enzyme production capacity of the cells was investigated. As Fig. 5 demonstrates, the *M. andauensis* cells have a wide range of extracellular enzyme activity. The cells produced proteases capable of clearing casein (Fig. 5A) and melting gelatin (Fig. 5B) in contrast to *S. cerevisiae* cells used as control, which did neither. Furthermore, a test carried out from a cell-free supernatant demonstrated the presence of a BSA-degrading enzyme in a chromatographic fraction (sample 3, Fig. 5C). Further tests demonstrated the production of acid (Fig. 5D), amylase (Fig. 5E), β-glucosidase production capacity (Fig. 5F). Besides, the cells were able to reduce the intensity of the Congo red staining reaction applied to a carboxymethyl-cellulase containing medium (Fig. 5G).

## Discussion

Microbes found on the surface of plants and fruits can be harmless or can cause spoilage of fruits or health problems^2,3^. To inhibit the growth of these harmful microbes, different approaches can be used, ranging from the application of synthetic chemicals to the use of environment-friendly microorganisms to effect biological control^5,24,25^. *Metschnikowia andauensis* belongs to these micoorganisms, as it is able to control growth of different microbes effectively^20,22,26^.

To expand the list of species whose growth can be inhibited by *M. andauensis*, wild yeasts isolated from fruit and flowers were investigated for their susceptibility to *M. andauensis*. The strains were identified in this or previous studies^27–30^. The strains included nine *Candida* species, *Pichia dorogensis* and *P. kluyveri, Issatchenkia terricola* and *Starmerella caucasica*. *M. andauensis* was able to inhibit the growth of *Starmerella caucasica, Pichia dorogensis, Issatchenkia terricola* strains and several *Candida* species, including *Candida orthopsilosis* and *C. butyri,* which are of medical importance^23,31^. We also confirmed the antagonistic effect of *M. andauensis* against the postharvest pathogen *Botrytis cinerea*. Taking into account other previously reported susceptible species^20,22,26^, the *M. andauensis* emerges as having a wide spectrum of antimicrobial activity.

To elucidate the processes which contribute to its inhibitory capacity, we confirmed that this species produces pulcherrimin, as do *M. pulcherrima* and, unexpectedly, *M. chrysoperlae.* The latter species was previously reported not to be capable of pigment production and it was initially used here as a negative control^11,12,17,18,21,32^. To confirm the identity of the pigment produced by *M. andauensis*, we isolated and characterized it by UV–Vis spectroscopy and FAAS analyses. The UV–Vis spectrum of the *M. andauensis* pigment showed the same characteristics as the pulcherrimin made by *M. pulcherrima*. The visible light absorption spectrum is typical of the presence of metal coordination or a conjugated electron pair system in this compound^13^*.* The FAAS analysis demonstrated the presence of iron in the pigment of *M. andauensis*. It is therefore reasonable to conclude that *M. andauensis,* like *M. pulcherrima* and *Bacillus subtilis*, can produce pulcherrimin and that iron coordination is responsible for the reddish colour^13,33^.

The production and extent of the pulcherrimin diffusion zone was strongly influenced by the composition of the medium. These data are in good agreement with what has been found for *M. pulcherrima*^8,11,34^. The size and colour intensity of the reddish zones were increased by the use of galactose, or disaccharides, such as maltose, trehalose, cellobiose, sucrose as carbon sources, addition of pectin, or higher concentrations of certain sugars. Interestingly, glucose and fructose stimulated growth but not pigment production of *M. andauensis* after a longer incubation time (13 days), which led to the formation of larger colonies that overran the pigmented zones that had appeared after 3 days.

Supplementation of media with amino acids, such as leucine, glutamic acid, arginine or others also favoured pigment synthesis. Since similar results were obtained with *Bacillus subtilis,* this data and existence of genes with similar functions found in yeasts and bacteria suggest similar pathway for synthesis of pulcherrimin^12,16,17,33^. Our further experiments showed that the size of the red zone on agar is pH dependent. The size increased at higher pH (pH 6.0–7.0) at all temperatures (25, 30, 37 °C) and decreased at lower pH (pH 4.0–5.5) or at higher iron concentrations (0.05, 1 mg/ml). As pH influences the ratio of ferric (Fe3^+^) and ferrous (Fe2^+^) ions^35,36^, it also affects the availability and uptake, and is expected to have a strong impact on migration of the precursor and the amount of pulcherrimin produced.

We do not know whether the larger and mostly lighter red or the narrower, dark red pigmented zones has the stronger antagonistic effect. The darker colour pigment intensity might indicate that a higher amount of iron is bound, which can cause a higher iron depletion from the environment and thereby growth inhibition of the microbes. The larger pigmented zone appears to cause a more extensive inhibition, as evidenced by the observation that borders of the red pigmented and inhibitory zones are often correlated. Since the iron-containing pulcherrimin is regarded as a water-insoluble and non-diffusible complex^8,13–15^, while the precursor molecule is thought to be a diffusible agent^37,38^, it is possible that the precursor can also have an inhibitory effect. This might be supported by our observation that the synthetic cyclo-leucyl–leucyl could inhibit yeast growth. It is not clear whether cyclo-leucyl-leucyl is found in the extracellular region of *M. andauensis* in nature, although the closely related *M. pulcherrima* does secrete cyclo-leucyl–leucyl^12^. In any case, this result is in good agreement with the findings which show that highly stable cyclic dipeptides can have antifungal, antibacterial effects^39–41^.

Since production of the pulcherrimin occurs even in the presence of 0.05 mg/ml copper sulphate, which is widely used in agriculture because of its fungicidal and bactericidal properties, the red pigment and/or its precursor should function as biocontrol agents in the field, even in the presence of agricultural copper sulphate.

Our results also revealed that the *M. andauensis* cells could produce proteases, as suggested by the degradation of casein, BSA and gelatin. To demonstrate a relationship between protease production and antifungal capacity requires further studies, but it is plausible, as a positive correlation has been found between protease activity and biocontrol capacity in certain bacterial strains^42^. Further studies showed that *M. andauensis* produces acids, and might have amylase and β-glucosidase activities. However, production of certain enzymes may depend on composition of the culture medium, as protease production and starch utilization has not been detected on other media^20–43^. At the same time, its β-glucosidase activity is in good agreement with the previous results^22^. This latter capacity might also contribute to appearance of the lighter red zone around the cells on the carboxymethyl-cellulose containing medium. All these processes, together or separately, may contribute to the wide-spectrum antifungal ability of *M. andauensis*.

In conclusion, the present study gives further evidence for the antagonistic capacity of the pigment producer *M. andauensis* and that this yeast species produces iron-chelate pulcherrimin. We demonstrated that there is a correlation between antagonistic capacity and the size of the red pigmented zone and determined some environmental factors that can increase the amount of pulcherrimin produced. Our results show that this species is able to produce proteases, α-glucosidase and amylase, which may contribute to the antagonistic capacity of *M. andauensis.* All these data suggest that this species has a great potential as a biocontrol agent. These data can contribute to the process of optimizing its efficacy in field trials and selecting the proper plant material.

## Material and methods

### Strains, culture media and taxonomic identification

#### Strains and their origin

The strains used in this study and their origin are listed in Table 1. Three *Metschnikowia* collection strains—two pigment-producing: *M. pulcherrima* (11-11, 11-578) and *M. crysoperlae* (11-1158) strain, which was chosen to be a negative control, but was found in the study to produce a small amount of pigment under certain conditions—and one *Saccharomyces cerevisiae* (11-642) were used as reference material. The susceptibility of 19 wild yeast strains which were isolated from fruits or flowers was tested to *M. andauensis*. They were collected in different geographical regions (Table 1). The samples were immersed in sterile water and aliquots were spread onto YDP medium. After 7 days single yeast colonies were isolated.

A *B. cinerea* strain (1) was also used to test antagonistic activities of the *M. andauensis* (11-1120) strain.

#### Identification of wild yeasts

PCR and sequencing methods were applied for determination of taxonomic positions of the collected yeast strains. Sequences of the D1/D2 domains of the large subunit ribosomal RNA gene were amplified by PCR (primers: NL1-5′-GCA TAT CAA TAA GCG GAG GAA AAG-3′ and NL4-5′-GGTCCG TGT TTC AAG ACG G-3′)^49^. The reaction was subjected to the following program: 94 °C 2 min, 95 °C 1 min, 51 °C 1 min 72 °C 1 min, (30X). The PCR fragments were purified and sequenced using the same primers. The taxonomic positions of the strains were accepted when 100% identity was found in the BLAST analysis to sequences of the type strains (NCBI database-https://blast.ncbi.nlm.nih.gov/Blast).

#### Culture media

Compositions of the media and culture conditions are listed in Table S1. YDP medium was used for the strain isolation, while YEA was generally used as standard medium.

Antimicrobial activity of *M. andauensis* against wild yeasts was tested on SMA medium incubated at 25 and 37 °C. Presence of the inhibitory zone was checked daily.

Pigment production and its diffusion were tested on different media, such as the complete medium YDP, Synthetic Minimal Media SML, SMA^44^ and Edinburgh Minimal Medium **(**EMMA)^45^, Potato Dextrose Media (PDA and PDB) and their modified versions (Table S1). To increase the visibility of the pulcherrimin ring the media were generally supplemented with FeCl_3._ Colour intensity and size of the red pigmented zone were checked daily.

SMG (gelatin-containing medium) was used to evaluate protease activity. SMG media were prepared with different brands of gelatin (Oetker or Lucullus). Since 2 and 4% gelatin plates were softer than 2% agar plates, *M. andauensis* cells were dropped and not streaked onto the surface of the plates, which were incubated at 24 °C and melting of the medium around the yeast cells was checked daily.

Media for extracellular enzyme activity assays were prepared according to the previous articles using pH5 and 7 values^46–48^ (Table S1). Protease activity was tested on casein-containing medium^46^, while amylase activity was tested on starch-containing rich medium^46^. A possible β-glucosidase activity was tested on cellobiose-containing rich medium^47^. Acid productivity was tested on CaCO_3_-containing medium^48^, while cellulose degradation was tested on carboxymethyl-cellulose-containing rich medium^46^.

*Botrytis cinerea* cultures were grown on YM medium. Antagonistic capacity of *M. andauensis* against *Botrytis cinerea* was tested on SMA and SMA supplemented with different amount of CuSO_4_. Presence of inhibitory zone was checked daily.

### Test for antimicrobial activity against yeasts and *Botrytis cinerea*

#### Spot assay to monitor the antimicrobial activity of *M. andauensis* cells against yeasts

The *M. andauensis* cells (11-1120) (test-strain) were cultured in a shaker in YEL medium at until OD_595_ = 1. The cells were harvested, washed with sterile distilled water and a cell suspension was prepared in sterile water. 15 ul of a cell suspension containing 7 × 10^7^ cells/ml was dropped onto the surface of the SMA media (pH5 and pH7) which had earlier been flooded with 400 µl cell suspension of the strain used as lawn (OD_595_ = 1) and dried in a sterile box. Appearance of the inhibitory zone was checked daily. When the cells of the lawn were able to grow on the medium far from the test-strain, but not around it (that is, a clear inhibitory zone was found), inhibition was recorded as positive (see in the Table 2). In contrast, absence of an inhibitory zone was recorded as a negative (-) result. The results come from three or more separate experiments.

#### Test for antagonism against *Botrytis cinerea*

A small piece (5 mm x 5 mm) of a single colony of *Botrytis cinerea* (1) was cut out and moved into 150 ml YM medium (in a 250 ml Erlenmeyer flask) and incubated on a shaker. After 1 week, mycelia of the mold were ground by hand in a mortar and a weak suspension was made with sterilized water. 400 µl of the cell suspension was spread onto SMA and SMA supplemented with CuSO_4_. After drying, cells of the *M. andauensis* test-strain, a 15 μl inoculum containing 7 × 10^7^ cells/ml were dropped onto the agar plates. Growth of *Botrytis cinerea* around the test strain was checked daily. The results come from three separate experiments.

#### Isolation and characterisation of the red pigment produced by *M. andauensis*

##### Isolation of the red pigment

*M. andauensis* grown for one week at 30 °C in 50 ml of SML medium containing 0.01 mg/ml FeCl_3_. The culture was centrifuged at 9000 g at 4 °C for 20 min. The supernatant was collected and concentrated by lyophilization. The dry material was dissolved in 10 ml of 50 mM sodium acetate buffer (pH 5.0), and centrifuged at 13000 g at 4 °C for 40 min. The sample was filter-sterilized by passing through a sterile 0.22 μm membrane disk. The sample was fractionated using size-exclusion chromatography. One ml of the sample was loaded onto a Sephadex G-25 column (10 ml, d:8 mm) that was pre-equilibrated with 50 mM sodium acetate buffer, pH 5.0 and the eluate was collected in 1 ml fractions. All the fractions of the gel-filtered sample were subjected to UV–Vis spectrophotometry, SDS-PAGE analysis and a protease activity test.

All the UV–Vis spectra were collected on a Specord 210 Plus dual-beam spectrophotometer.

For the protease activity test, 20 μl of the chromatographic fractions was concentrated five times and incubated with 10 μl of a 5 mg/ml BSA solution for 6 h at 37 °C. The incubated solutions were analysed by 12% SDS-PAGE.

##### Study of the red pigment by Flame Atomic Absorption Spectrometry (FAAS)

The solid red pigment was weighed, and 65% nitric acid was added to produce iron (III) nitrate. After a few hours the supernatant was discarded, and the iron (III) nitrate was dissolved in MilliQ water. The concentration of Fe (III) ion was determined using the FAAS method (UNICAM SP 1900 AAS**)** with the following parameters: air: 4.6 l/min (3 bar), acetylene: 1.4 l/min, Narva iron hollow cathode lamp, optical slit width: 0.1 mm, lamp current: 15 mA, observation height: 10 mm, burner length slot: 10 cm.

#### Effect of environmental factors (C-source, presence of amino acid, pH, temperature or cell density) on pigment production and diffusion

##### Test for the effect of the C-source on pigment production

Different sugar-containing SMA media were prepared using 2% sugar concentration. To test the effect of sugar concentration, the media were prepared with 1, 2, and 4% sugar. All media were supplemented with 0.001 mg/ml FeCl_3._ The same amount of *M. andauensis* (11-1120) and *M. crysoperlae* (11-1158) cells were dropped onto the surface of the media. The plates were incubated at 25 °C and the pigmented zones were checked after 3 and 13 days.

The effect of pectin was investigated on YEA containing 0.5% or 1% pectin. The colour and size of the pigmented zone were compared to those of the agar plates prepared without pectin.

In order to learn the effect of agar concentration on the diameter of the pigmented zone, YDP containing 1 and 2.5% agar were used for culturing. The plates were measured after 2, 4, 6, 10 days.

##### Test for effect of amino acids on pigment production

A cell suspension was prepared from *M. andauensis* cells (11-1120) with sterile water. A 15 μl inoculum containing 1 × 10^6^ cells/ml was dropped onto the surface of PDA and SMA media (pH 7.0) supplemented with different amino acids and FeCl_3_. The results come from three separate experiments.

##### Relation between cell density and size of the red pigmented zone

Cell suspensions were prepared with sterile water from the *M. andauensis* (11-1120) cells grown on YEA for 1 day. A 15 μl inoculum containing 1 × 10^3^ or 1 × 10^7^ cells/ml was dropped onto the surface of SMA (pH 7.0) and PDA (pH 7.0) supplemented with 0.01 mg/ml FeCl_3_. The agar plates were incubated at 25 °C. After 5 days size of the red pigmented zone was measured by ruler. The results come from three separate experiments.

#### Investigation of extracellular enzyme activity and growth of gelatin medium

##### Extracellular enzyme activity assays

*M. andauensis* (11-1120) and *M. pulcherrima* (11-578) cells were grown on YEA for 3 days at 25 °C. Cell suspensions were preapared with sterile distilled water, whose cell densities were OD_595_ = 0.1. 15 µl of the cell suspensions were dropped onto the surface of the given media. The agar plates were incubated at 25 °C. The protease activity was tested on casein-containing medium. Production of protease was indicated by the presence of a clear zone around the colony^46^. Amylase activity was tested on starch-containing rich medium, after washing the plates with lugol solution. A clear zone was observed around the colony when the cells had enzymatic activity^46^. Cellulose degradation capacity was tested on carboxymethyl-cellulose-containing medium. Activity of the cellulose degrading enzymes is indicated by appearance of a transparent halo around the colony when washed with Congo Red^46^. a possible β-glucosidase activity was tested on cellobiose-containing rich medium^47^. Acid productivity was indicated by a clear zone around the cells on CaCO_3_-containing medium^48^.

##### Growth on gelatin solidified medium

To obtain further evidence for protease activity, growth on gelatin medium was also tested. *M. andauensis* (11-1120) and non pigment-producing *Saccharomyces cerevisiae* (10-642) were cultured in YPL medium at 28 °C in a shaker. After 1 day, a 10 µl cell suspension containing 1.5 × 10^6^ cells/ml was dropped onto the surface of the media solidified with gelatin. The agar plates were incubated at 24 °C and melting of the medium around the *M. andauensis* cells was investigated.

#### Preparation and investigation of cyclo-leucyl-leucyl

##### Preparation of cyclo-leucyl-leucyl

N,N′-Dicyclohexyl-carbodiimide (1.05 g, 4.30 mmol) was added to the solution of Leu-Leu dipeptide (PubChem) in N,N-dimethylformamide (50 ml) and stirred for 30 min, then pyridine (2 ml) was added and the reaction mixture was stirred for 24 h. The reaction mixture was concentrated and co-concentrated with toluene (3 × 20 ml). Column chromatography (hexane:ethyl acetate 7:3) of the residue afforded the capture of the cyclic product (880 mg, 77%). The concentrated cyclic product was dissolved in DMSO (Sigma, final concentration was 100 mg/ml). The cyclic-leucyl-leucyl solution was divided into Eppendorf tubes (100–100 µl) and stored in − 20 °C.

##### Effect of cyclo-leucyl-leucyl on growth of yeasts

Cell suspensions of *Saccharomyces cerevisiae* (11-481, 10-462), *Candida stigmatis* (11-465), or *Starmerella caucasica* (11-1071) were prepared with sterile water from 1-day-old cells grown on YEA at 25 °C. The final cell density of the cell suspensions was OD_595_ = 1 and the surface of the YEA plates was flooded with the cell suspensions to form a lawn. After drying under a sterile box, a 15 µl inoculum containing 100 mg/ml cyclo-leucyl-leucyl dissolved in DMSO (Sigma), two dilutions (50 and 33 mg/ml prepared with DMSO or PEG4000) or DMSO and PEG4000 as controls were dropped onto the surface of the media. The agar plates were incubated at 25 °C and growth of the lawn was investigated.

##### Statistical analysis

Statistical analysis was created using the BoxPlotR program (http://shiny.chemgrid.org/boxplotr/)^50^.

## Supplementary Information

Supplementary Information.

## Data Availability

The dataset(s) supporting the conclusions of this article is (are) included within the article.

## References

[1] Chan Z, Tian S (2005). Interaction of antagonistic yeasts against postharvest pathogens of apple fruit and possible mode of action. Postharvest Biol. Technol..

[2] Zhimo VY, Dilip D, Sten J, Ravat VK, Bhutia DD, Panja B, Saha J (2017). Antagonistic yeasts for biocontrol of the banana postharvest anthracnose pathogen *Colletotrichum musae*. J. Phytopathol..

[3] Dukare AS, Sangeeta PV, Nambi E, Gupta RK, Singh R, Sharma K, Vishwakarma RK (2019). Exploitation of microbial antagonists for the control of postharvest diseases of fruits: a review. Crit. Rev. Food Sci. Nutr..

[4] Ghorbanpour M, Omidvarib M, Abbaszadeh-Dahajic P, Omidvard R, Kariman K (2018). Mechanisms underlying the protective effects of beneficial fungi against plant diseases. Biol. Control.

[5] Heydari A, Pessarakli M (2010). A review on biological control of fungal plant pathogens using microbial antagonists. J. Biol. Sci..

[6] Neilands JB (1995). Siderophores: structure and function of microbial iron transport compounds. J. Biol. Chem..

[7] Philpott CC (2006). Iron uptake in fungi: a system for every source. Biochem. Biophys. Acta..

[8] Roberts C (1946). The effect of iron and other factors on the production of pigment by the yeast *Torulopsis pulcherrima*. Am. J. Bot..

[9] Raspor P, Miklic-Milek D, Avbelj M, Cadez N (2010). Biocontrol of *B. cinerea* with wine yeasts, biocontrol of grey mould disease on grape caused by botrytis cinerea with autochthonous wine yeasts. Food Technol. Biotechnol..

[10] Oro L, Ciani M, Comitini F (2014). Antimicrobial activity of *Metschnikowia pulcherrima* on wine yeasts. J. Appl. Microbiol..

[11] Sipiczki M (2006). *Metschnikowia* Strains isolated from botrytized grapes antagonize fungal and bacterial growth by iron depletion. Appl. Environ. Microbiol..

[12] Gore-Lloyd D (2019). *Snf2* controls pulcherriminic acid biosynthesis and antifungal activity of the biocontrol yeast *Metschnikowia pulcherrima*. Mol. Microbiol..

[13] Kluyver AJ, van der Walt JP, van Triet AJ (1953). Pulcherrimin, the pigment of *Candida pulcherrima*. Botany.

[14] MacDonald JC (1965). Biosynthesis of pulcherriminic acid. Biochem. J..

[15] Cook AH, Slater CA (1956). The structure of pulcherrimin. J. Chem. Soc..

[16] Krause DJ (2018). Functional and evolutionary characterization of a secondary metabolite gene cluster in budding yeasts. Proc. Natl. Acad. Sci. USA.

[17] Sipiczki M (2020). *Metschnikowia pulcherrima* and related pulcherrimin-producing yeasts: fuzzy species boundaries and complex antimicrobial antagonism. Microorganisms.

[18] Molnar O, Prillinger H (2005). Analysis of yeast isolates related to *Metschnikowia pulcherrima* using the partial sequences of the large subunit rDNA and the actin gene; description of *Metschnikowia andauensis* sp. nov. Syst. Appl. Microbiol..

[19] Lachance, MA. *Metschnikowia kamienski (1899)* in *The Yeasts* (eds.) Kurtzman, C.P., Fell, J.W., Boekhout, T. **46**, 575–620 (2011).

[20] Manso T, Nunes C (2011). *Metschnikowia andauensis* as a new biocontrol agent of fruit postharvest diseases. Postharvest Biol. Technol..

[21] Manso, T., Vero, S., González, M.E., Nunes, C. Study of modes of action of the biocontrol agent *Metschnikowia andauensis* PBC-2. In: *Environmentally Friendly and Safe Technologies for Quality of Fruit and Vegetables.* (ed. Nunes, C.) 144–150 Universidade do Algarve, Faro, Portugal (2010).

[22] Pawlikowska E, Steve AJ, Breierova E, Antolak H, Kregiel D (2019). Biocontrol capability of local *Metschnikowia* sp. isolates. Antonie Van Leeuwenhoek.

[23] Arastehfar, A., et al. Molecular identification, genotypic diversity, antifungal susceptibility and clinical outcomes of infections caused by clinically underrated yeasts, *Candida orthopsilosis* and *Candida metapsilosis*. An Iranian multicenter study (2014–2019). *Front. Cell. Infect. Microbiol.* **(9),** article 264 (2019). 10.3389/fcimb.2019.00264 PMC6682699 31417877

[24] Ray RC, Swain MR, Panda SH, Singh A (2011). Microbial control of postharvest diseases of fruits, vegetables, roots, and tubers. Bioaugmentation, Biostimulation and Biocontrol Soil Biology.

[25] Mohd JJ, Dar NA, Bhat TA, Bhat AH, Bhat MA (2013). Commercial biocontrol agents and their mechanism of action in the management of plant pathogens. Int. J. Mod. Plant Anim. Sci..

[26] Horváth, E., Sipiczki, M., Csoma, H., Miklós, I. Assaying the effect of yeasts on growth of fungi associated with disease. *BMC Microbiol.* **20,** article number 320 (2020). 10.1186/s12866-020-01942-0 PMC7579944 33087058

[27] Sipiczki M (2010). Candida stigmatis sp. nov., a new anamorphic yeast species isolated from flowers. FEMS Yeast Res.

[28] Sipiczki M (2013). Detection of yeast species also occurring in substrates associated with animals and identification of a novel dimorphic species in *Verbascum* flowers from Georgia. Antonie Van Leeuwenhoek.

[29] Sipiczki M (2013). *Starmerella caucasica* sp. nov., a novel anamorphic yeast species isolated from flowers in the Caucasus. J. Gen. Appl. Microbiol..

[30] Sipiczki M (2014). *Metschnikowia laotica* f.a., sp. Nov., a dimorphic, pigment-producing yeast species isolated from fruit. Int. J. Syst. Evol. Microbiol..

[31] Nakase T (1971). Four new yeasts found in Japan. *C. butyri*, may also be found in human samples as opportunists awaiting the proper change in environmental conditions to become pathogens, e.g., in immunocompromised hosts. J. Gen. Appl. Microbiol..

[32] Suh SO, Gibson CM, Blackwell M (2004). *Metschnikowia chrysoperlae* sp. nov., *Candida picachoensis* sp. nov. and *Candida pimensis* sp. nov., isolated from the green lacewings Chrysoperla comanche and *Chrysoperla carnea* (Neuroptera: Chrysopidae). Int. J. Syst. Evol. Microbiol..

[33] Uffen RL, Canale-Parola E (1972). Synthesis of pulcherriminic acid by *Bacillus subtilis*. J. Bacteriol..

[34] Melvydas V, Staneviciene R, Balynaite A, Vaiciuniene J, Garjonyte R (2016). Formation of self-organized periodic patterns around yeasts secretinga precursor of a red pigment. Microbiol. Res..

[35] Kosman DJ (2013). Iron metabolism in aerobes: managing ferric iron hydrolysis and ferrous iron autoxidation. Coord. Chem. Rev..

[36] Sánchez M, Sabio L, Gálvez N, Capdevila M, Dominguez-Vera JM (2017). Iron chemistry at the service of life. IUBMB Life.

[37] Grosbücsh J (1915). Uber eine farblose, stark roten Farbstoff erzeugende Torula. Centralbl. F. Bakt..

[38] Beijerinck MW (1918). Levures chromogenes-Nouvelle reaction biologique du fer. Arch Neerlandaises de Physiol de l'Homme et des Anim..

[39] Ström K, Sjögren J, Broberg A, Schnürer J (2002). *Lactobacillus* plantarum MiLAB 393 produces the antifungal cyclic dipeptides cyclo(L-Phe-L-Pro) and cyclo(L-Phe-trans-4-OH-L-Pro) and 3-phenyllactic acid. Appl. Environ. Microbiol..

[40] Mishra AK, Choi J, Choi SJ, Baek KH (2017). Cyclodipeptides: an overview of their biosynthesis and biological activity. Molecules.

[41] de Andrade VM (2020). Antifungal and anti-biofilm activity of designed derivatives from kyotorphin. Fungal Biol..

[42] Wei LH, Xue QY, Wei BQ, Wang YM, Li SM, Chen LF (2010). Screening of antagonistic bacterial strains against *Meloidogyne incognita* using protease activity. Biocontrol Sci. Tech..

[43] Lachance MA, Bowles JM (2002). *Metschnikowia arizonensis* and *Metschnikowia dekortorum*, two new large-spored yeast species associated with floricolous beetles. FEMS Yeast Res..

[44] Sipiczki M, Ferenczy L (1978). Enzymic methods for enrichment of fungal mutants. Enrichment of *Schizosaccharomyces pombe* mutants. Mutat. Res..

[45] Mitchison JM (1970). Physiological and cytological methods for *Schizosaccharomyces pombe*. Methods Cell Physiol..

[46] Ganga MA, Martinez C (2004). Effect of wine yeast monoculture practice on the biodiversity of non-*Saccharomyces* yeasts. J. Appl. Microbiol..

[47] Villena AM, Iranzo JFÚ, Perez AIB (2007). β-Glucosidase activity in wine yeasts: application in enology. Enzyme Microb. Technol..

[48] Kreger-van R (1984). The Yeasts: A Taxonomic Study.

[49] O’Donell, K. Fusarium and its near relatives. In: *The Fungal Holomorph: Mitotic, Meiotic and Pleomorphic Speciation in Fungal Systematics*, (eds) Reynolds, D.R., Taylor, J.W. 225–233, CAB International, Wallingford, UK (1993).

[50] Potter, K. Methods for Presenting Statistical Information: The Box Plot. In: *Visualization of Large and Unstructured Data Sets, GI-Edition Lecture Notes in Informatics (LNI),* (eds.) Hagen, H., Kerren, A., Dannenmann, P. S-4, 97–106, (2006).

