# Supplementary material for: The antagonistic Metschnikowia andauensis produces extracellular enzymes and pulcherrimin, whose production can be promoted by the culture factors
Source: Sci Rep. 2021 May 19;11:10593. doi: 10.1038/s41598-021-89982-y (PMC8134588; doi:10.1038/s41598-021-89982-y)
Supplement: Supplementary file 1 — Supplementary Information. [file 41598_2021_89982_MOESM1_ESM.docx]

Table S1. Composition of the media and culturing conditions

| **Medium** | **Composition** | **Culturing conditions** | **Application** | **Reference** |
| --- | --- | --- | --- | --- |
| **Basic media** | | | | |
| YDP | 1% yeast extract  2% glucose  2% peptone  2% agar | 25^o^C  7 days | Strain isolation |  |
| YEA | 1% yeast extract  2% glucose  2% agar | 25^o^C  1 day | Culturing of yeast cells (standard medium) |  |
| YEL | 1% yeast extract  2% glucose | 25^o^C  1 day | Culturing of yeast cells |  |
| SMA | SML+2% agar |  |  | [44] |
| EMMA |  |  |  | [45] |
| PDA, PDB |  |  |  | Scharlau  01-483-50  02-483-500 |
| YM | 0.3% malt extract  0.3% yeast extract 0.5% peptone  1% glucose | 22^o^C  1 week | Culturing of *B. cinerea* mycelia |  |
| **Media for testing of extracellular enzyme activity** | | | | |
| Casein-containing medium | 0.3% yeast extract  0.3% malt extract 1.5% casein  0.5 % pepton  2% agar | 25^o^C  1-6 days | Test of protease activity | [46] |
| SMG | SMA solidified with 2 and 4% gelatin instead of agar  pH4.5-5 | 24^o^C  1-6 days | Test of protease activity | This study |
| Starch-containing-medium | 1.5% pepton  1% yeast extract 1% starch  2% agar | 25^o^C  1-6 days | Test of amylase activity | [47] |
| Carboxymethyl-cellulose-containing medium | 0.3% pepton  0.3% yeast extract  1% carboxy-methyl- cellulose (Sigma)  2% agar | 25^o^C  1-6 days | Test of cellulase activity | [46] |
| Cellobiose-containing medium | 1% yeast extract 2% pepton  2% cellobiose  2% agar | 25^o^C  1-6 days | Test of β-glucosidase activity | [47] |
| CaCO_3_–containing medium | 0.5% yeast extract  5% glucose  2% agar  0.5% CaCO_3,_ pH5 | 25^o^C  1-6 days | Test of acid productivity | [48] |
| **Modified media for testing of environmental factors** | | | | |
| SMA |  | supplemented with 0.0005, 0.005 and 0.05 mg/ml CuSO_4_  25^o^C  5days | Antagonistic capacity against *B. cinerea* in presence of CuSO_4_- |  |
| SMA | SML+2% agar | supplemented with 0.001 mg/ml FeCl_3_ and containing  2% galactose  or 2%mannose  or 2% fructose  or 2% maltose  or 2% trehalose or 2% cellobiose or 2% sucrose instead of glucose  25^o^C  3-13 days | Test for effect of the C-source on pigment production | [44] |
| YEA |  | supplemented with 0.005 mg/ml FeCl_3_  plus 0.5 and 1% pectin  15, 25, 30^o^C  1-15 days  pH5 | Effect of pectin concentration on pigment production |  |
| SMA,PDA |  | supplemented with 0.005 mg/ml FeCl_3_ and with leucine  or glutamic acid or arginine  or lysine  or serine  or threonine  or alanine (amino acid concentrations were: 0.02, 0.20, 2.00 mg/ml)  25^o^C  6 days  pH7 | Effect of amino acids on pigment production |  |
| SMA | supplemented with 0.05 mg/ml FeCl_3_ or 0.05 mg/ml Cu SO_4_  or 0.05 mg/ml CuSO_4_ plus 0.05 mg/ml FeCl_3_ | 25^o^C  11 days | Budding of *M. andauensis* cells in the presence of CuSO_4_ | [44] |
